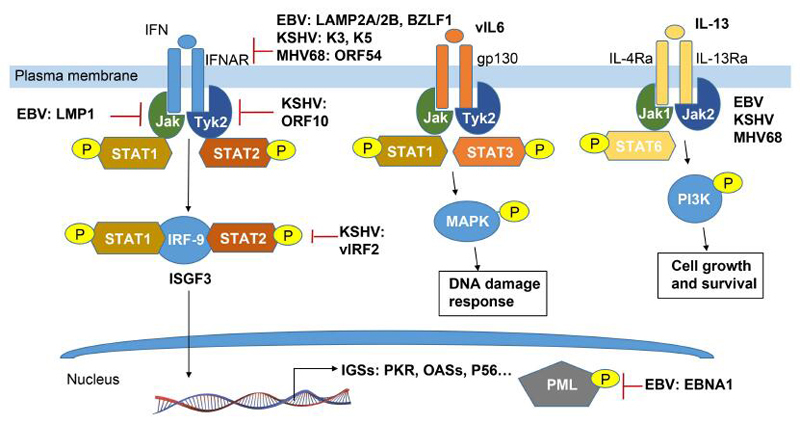# The regulatory role of protein phosphorylation in human gammaherpesvirus associated cancers

**DOI:** 10.1007/s12250-017-4081-9

**Published:** 2017-10-30

**Authors:** Yuyan Wang, Shuvomoy Banerjee, Ling Ding, Cankun Cai, Fang Wei, Qiliang Cai

**Affiliations:** 1 MOH & MOE Key Laboratory of Medical Molecular Virology, School of Basic Medical Sciences, Shanghai Medical College of Fudan University, Shanghai, 200032 China; 2 Amity Institute of Virology and Immunology, Block-J3, Sector-125, Amity University, Uttar Pradesh, 201303 India; 3 Sheng Yushou Center of Cell Biology and Immunology, School of Life Sciences and Biotechnology, Shanghai Jiao Tong University, Shanghai, 200240 China

**Keywords:** Epstein-Barr Virus (EBV), Kaposi’s sarcoma-associated herpesvirus (KSHV), phosphorylation

## Abstract

Activation of specific sets of protein kinases by intracellular signal molecules has become more and more apparent in the past decade. Phosphorylation, one of key posttranslational modification events, is activated by kinase or regulatory protein and is vital for controlling many physiological functions of eukaryotic cells such as cell proliferation, differentiation, malignant transformation, and signal transduction mediated by external stimuli. Moreovers, the reversible modification of phosphorylation and dephosphorylation can result in different features of the target substrate molecules including DNA binding, protein-protein interaction, subcellular location and enzymatic activity, and is often hijacked by viral infection. Epstein-Barr virus (EBV) and Kaposi’s sarcomaassociated herpesvirus (KSHV), two human oncogenic gamma-herpesviruses, are shown to tightly associate with many malignancies. In this review, we summarize the recent progresses on understanding of molecular properties and regulatory modes of cellular and viral proteins phosphorylation influenced by these two tumor viruses, and highlight the potential therapeutic targets and strategies against their related cancers.

## INTRODUCTION

Protein phosphorylation is a reversible post-translational modification (PTM) that occurs at specific residues as serine, threonine, tyrosine, and involves a series of sequence-specific kinases, phosphatases and recognized proteins. Phosphorylation signaling is a critical process for its ability to modulate cellular protein activities, alter protein folding, and initiate or inhibit interactions with other proteins. Importantly, phosphorylation plays a significant role in formation and development of cancer through alteration of oncogenic kinase signaling (Smith et al., 2012), transcriptional regulation (Morin et al., 1997), and TP53 activity (Liu et al., 2004). The reversible phosphorylation of proteins regulates almost all aspects of cell life cycle, while abnormal phosphorylation is a cause or consequence of many diseases. It has been demonstrated that mutations in particular protein kinases and phosphatases give rise to a number of disorders, and many naturally occurring toxins and pathogens exert their effects by altering the phosphorylation states of intracellular proteins (Cohen, 2001). This includes amino acid substitutions on kinases or phosphatases that directly interrupt the stability and/or the function of the kinase or phosphatase, resulting in changes of target protein phosphorylation. The regulators of kinase or phosphatase can also indirectly play an effect on alteration of target protein phosphorylation (Stephens et al., 2005). Interestingly, increasing evidence has shown that disruptions of phosphorylation sites of key proteins are associated with many cancers (Alt et al., 2000), and phosphorylation can be pharmacologically targeted with multiple approved therapies for cancer treatment (Tiacci et al., 2011). For instance, loss of phosphorylation is causatively implicated in nuclear accumulation of cyclin D1 in esophageal cancer and generally increased malignancy potential (Benzeno et al., 2006). Another example shows that activation of Akt kinase by phosphorylation is tightly associated with cell-cycle progression (Liu et al., 2014). Thus, phosphorylation events are highly potential target to be invaded by viral infection for viral replication and propagation.

Epstein-Barr virus (EBV, or human herpesvirus-4, HHV-4) and Kaposi's sarcoma-associated herpesvirus (KSHV, or human herpesvirus-8, HHV-8), two members of the gamma-herpesvirus subfamily of human herpesviruses, are double-stranded DNA tumor viruses with genome size range within 100 to 200 kbp, and are considered as the major contributors to lymphomagenesis in the immune-deficient humans. Importantly, these two gamma-herpesviruses are accountable for several lymphoproliferative and neoplastic disorders. For examples, EBV is etiologically associated with infectious mononucleosis, Burkitt's lymphoma (BL), nasopharyngeal carcinoma (NPC), Hodgkin's disease, hemophagocytic lymphohistiocytosis syndrome and some gastric cancers (Jha et al., 2016). In contrast, KSHV is associated with Kaposi's sarcoma (KS), and primary effusion lymphoma (PEL) and multicentric Castleman's disease (MCD) (Parravicini et al., 2000). Upon viral infection, it has been shown that a number of cellular signaling pathways involving in phosphorylation and dephosphorylation events are stimulated (Brinkmann and Schulz, 2006). This review will focus on the human oncogenic herpesviruses EBV and KSHV, and address the recent progress on how these two tumor viruses hijack the cellular protein phosphorylation signaling for gene expression, cell growth and immune escape, as well as highlight their potential therapeutic targets and strategies against the viral related cancers.

## VIRAL PROTEINS IS PHOSPHORYLATED BY CELLULAR AND VIRAL KINASES

The phosphorylation of target proteins induced by viral infection has been shown to play major impacts on viral invasion, replication, and cytotoxicity of the host cells. The addition or removal of a negatively charged phosphate group by kinases or phosphatases has been reported to regulate target protein's stability, activity as well as interactions with other cellular and viral proteins (Jakubiec and Jupin, 2007). Once specific phosphorylation sites of viral proteins are identified, mutational analyses will reveal the potential phenotypic effects of such viral protein's phosphorylation site. In many cases, multiple kinases are able to phosphorylate the same protein. Henceforth, by targeting different kinase profiles, a virus could have the ability to expand its host and cellular tropism, and to infect different species and cell types. On the other hand, kinase redundancy also provides multiple opportunities for a viral protein to be phosphorylated, ensuring the chance for the phosphorylated protein to induce pathogenic effects on the cell. The phenomenon of kinase redundancy has been found to exist in a number of viruses including EBV and KSHV. For example, both the SM protein (an early stage protein of EBV lytic replication) and latent membrane protein LMP1 can be phosphorylated by casein kinase II (CKII) (Cook et al., 1994; Chi et al., 2002). During cell mitosis, the latent antigen EBNA2 is phosphorylated at Ser 243 by cdc2/cyclin B1 kinase for activation of the Cp promoter within EBV genome (Yue et al., 2006). Interestingly, the EBV nuclear antigen leader protein EBNA-LP is not only activated by cellular kinase cdc2 but also viral kinase BGLF4 for phosphorylation at Serine 35 (Kato et al., 2003). LMP2A is able to induce phosphorylation at Ser 15 and Ser 102 *in vitro* by mitogen-activated protein kinase MAPK in the control of viral latency (Panousis and Rowe, 1997). Although tyrosine phosphorylation of LMP2A occurs in both B lymphocyte and epithelial cells, the CSK (a negative Src regulator) instead of Src family kinase (LCK, Lyn and FYN) is shown to phosphorylate LMP2A in epithelial cells (Burkhardt et al., 1992; Scholle et al., 1999). In contrast, in regard to how KSHV utilizes cellular kinase to modulate its own proteins, it has been found that K-bZIP (expressed during lytically infected B cells) was phosphorylated on Thr 111 and Ser 167 by CDK1 and CDK2, respectively (Polson et al., 2001). Moevoer, the latent protein Kaposin B is phosphorylated by activation of p38 MAPK for induction of proinflammatory cytokines and blocking cytokine mRNA decay (McCormick and Ganem, 2006).

In addition to utilize cellular kinases, gamma-herpesviruses also encode their own kinases to phosphorylate target proteins (Asai et al., 2006). Interestingly, most of viral kinases not only phosphorylate other viral and cellular proteins, but also autophosphorylated themselves, which facilitates viral replication or production within the host cells (Kawaguchi and Kato, 2003). For instance, the EBV-encoded BGLF4 is a protein kinase, and is able to phosphorylate a number of viral proteins including BZLF1 (Asai et al., 2006), EA-D (Chen et al., 2000), and EBNA2 during the latency (Kato et al., 2003). Surprisingly, during the viral lytic replication, BGLF4 also phosphorylates EBNA-2 in a manner similar to the cellular kinase cdk1, and the hyperphosphorylation of EBNA-2 will in turn inhibit its normal ability to transactivate the EBV LMP1 promoter, and eventually lead to induction of EBV lytic replication (Yue et al., 2005). This indicates it is not absolutely that phosphorylation could have clear-cut effects on viral life cycles. Similarly, KSHV encoded ORF36 protein is also a serine protein kinase and is able to inhibit cell spreading and FAK activation through interacting with FAK and blocking its tyrosine phosphorylation (Park et al., 2000; Park et al., 2007).

Autophosphorylation of cellular kinases can occur intermolecularly or intramolecularly, which will potentially alter the protein conformation, and positively or negatively regulate the catalytic activity of the kinase (Wang and Wu, 2002; Pickin et al., 2008). In addition, phosphorylation of a target kinase can also influence its interaction with other proteins. For example, tyrosine phosphorylation of specific residues within Src-family kinases is required for Src to interaction with proteins carrying SH2 domains (Pawson, 1995; Thomas and Brugge, 1997). For herpesvirus, in addition to HCMV UL97 (He et al., 1997) and HSV-1 UL13 (Cunningham et al., 1992), EBV encoded BGLF4 has also presented an ability to autophosphorylate itself (Kato et al., 2001), albeit the full effects of these autophosphorylations on protein activity remains to be further investigation. Moreover, it is still unknown whether autophosphorylation of viral kinases will induce the same effect, such as regulating catalytic activity and recruiting other proteins for interaction, as the autophosphorylation of cellular kinases.

## PHOSPHORYLATION EVENT REGULATES VIRAL TRANSCRIPTION AND TRANSLATION

In the view of the fact that animal viruses do not encode their own components of the translational machinery, it has been considered that the host translation factors play critical roles in viral pathogenesis. Viruses not only effectively modulate the array of factors required for polypeptide production, but also appropriately control the complex regulatory circuits that associate with their activity. Like so many other biological regulatory events, this is also achieved by altering the phosphorylation state of the target molecules. To regulate viral gene expression at transcriptional and post-transcriptional levels, ORF57 (or its counterpart) is encoded by most of herpesvirus (particularly KSHV), and is phosphorylated by CK2 in the presence of the complex with heterogeneous nuclear ribonucleoprotein K (hnRNP K) (Malik and Clements, 2004).

In addition, PKR is a serine/threonine kinase that involves in regulation of the phosphorylation of eukaryotic translation initiation factor 2 (eIF-2α). During viral latent infection, KSHV LANA2 blocks PKR-induced phosphorylation of eIF-2α to counteract the PKR-mediated inhibition of protein synthesis and apoptosis (Esteban et al., 2003). Furthermore, KSHV vIRF-2 also physically interacts with PKR and consequently inhibits its autophosphorylation and phosphorylation of PKR substrates of histone 2A and eukaryotic translation initiation factor 2 (eIF-2α) (Burysek and Pitha, 2001). Thus, the cooperation effect of vIRF-2 and LANA2 on PKR-mediated translational regulation may play a role in the viral maintenance and malignancy.

## PHOSPHORYLATION–MEDIATED VIRAL MANIPULATION OF DNA DAMAGE RESPONSE AND CELL PROLIFERATION

The DNA damage response (DDR) pathway has evolved to recognize viral DNA entering the nucleus of host cells during viral infection, which involve in phosphorylation of representative DDR-associated proteins, such as ATM and γH2AX (Figure 1). During *de novo* infection of primary endothelial cells, KSHV activates phosphorylation of ATM and H2AX for establishing viral latency (Singh et al., 2014). Further studies indicate that KSHV encoded LANA interacts with H2AX and induces phosphorylation

Figure 1. A schematic illustrating γ-herpesvirus hijacks DNA damage response to induce cell proliferation.

of γH2AX at serine 139 for episome persistence (Jha et al., 2013). Similarly, EBV also modulates the activation of DDR signaling during its lytic cycle, through Zta-mediated phosphorylation of ATM and γH2AX (Wang' ondu et al., 2015). However, overexpression of the latent proteins LMP1 and LMP2A encoded by EBV in the EBV negative NPC cells could cooperatively suppress activation of γH2AX phosphorylation at Serine 139 in response to genotoxic treatment (Wasil et al., 2015). Intriguingly, the acute infection of murine γ-herpesvirus 68 (MHV68) in myeloid cells markedly enhance phosphorylation of γH2AX. Further studies of the mutagenesis screening identified that the kinase ORF36 encoded by MHV68 is responsible to induce the γH2AX phosphorylation at Serine 139, and enhance viral replication by prolonging S phase (Tarakanova et al., 2007). Moreover, EBV but no other herpesvirus encoded ORF36 homolog (named BGLF4) display similar effect (Xie and Scully, 2007).

The oncogenic serine/threonine kinase of Pim-1/2/3 family has been shown to be highly upregulated in a number of human cancers. Among them, Pim-1 is able to phosphorylate itself, and there are several substrates have been identified, including p21, Cdc25A, NuMA for driving cell proliferation through the transition of G1/S and G2/M phase, and protecting cell from genotoxin-induced death (Pircher et al., 2000). In contrast, Pim-2 kinase is an essential component of the DNA-damage response, and is an upstream activator of the phosphorylation of pro-survival/anti-apoptotic factors E2F-1 and ATM (Zirkin et al., 2013). Overexpression of Pim-2 reduces γH2AX accumulation in DNA-damaged cells for exerting the protective effect. In the context of KSHV infection, the latent protein LANA is shown to activate Pim-1 and also act as a Pim-1 substrate (Bajaj et al., 2006), and the inactivation of LANA phosphorylation at Serine residues 205 and 206 by Pim-1 and Pim-3 kinases is required to trigger induction of KSHV lytic replication (Cheng et al., 2009). Upon EBV latent infection, not only Pim-1 is required for LMP1-induced cell survival, but both Pim-1 and Pim-2 are also upregulated, and in turn enhance the activity of EBNA2 in driving EBV-induced cell immortalization (Rainio et al., 2005; Kim et al., 2010). Further studies reveal that other latent antigen EBNA3C interacts with and stabilizes Pim-1, which leads to Pim-1-mediated phosphorylation of the Cyclin inhibitor p21 at the threonine 145 residue for promoting B-cell proliferation (Banerjee et al., 2014). Surprisingly, inhibition of ATM/ChK2 instead of ATR/Chk1 will markedly increase EBV-transformation efficiency of primary B cells (Nikitin et al., 2010; Mordasini et al., 2017).

## PHOSPHORYLATION–MEDIATED VIRAL ESCAPE OF HOST INNATE IMMUNE RESPONSE

In response to viral infection, the host innate immune system is triggered. It is well now known that many viral components can be initially sensed by the host innate pattern recognition receptors (PRRs), including the toll-like receptors (TLRs) and the RIG-I-like receptors (RLRs) to dsRNA, and Z-DNA binding protein 1 (ZBP1), absent in melanoma 2 (AIM2), or cGAMP synthase (cGAS) to free exogenous viral DNA in the cytosol. The PRR signaling pathways could activate some host adaptor proteins and lead to the induction of IFNs, proinflammatory cytokines and chemokines for controlling viral replication and spreading, even maturation and recruitment of the more specific adaptive immune response. During the activation of host immune responses, many viral components and cellular proteins are involved in regulation of phosphorylation events (Chen and Yuan, 2014; Chang et al., 2016). We will summarize and highlight how both EBV and KSHV modulate the phosphorylation events to escape the host innate immune response below (Figure 2).

### Phosphorylation-mediated sensing of viral components

The first step to trigger the host innate immune system is viral components sensed by PRRs, which includes glycoproteins and nucleic acids (such as dsRNA or CpG DNA), and then activating three main transcription factor complexes (IRF-3/IRF-7, NF-κB and ATF2/c-jun) involved in IFN production. In response to entry of DNA viruses, cytosolic exogenous double-stranded DNA (dsDNA) is recognized by and triggers both Z-DNA binding protein 1/DNA-dependent activator of IFN-regulatory factors (ZBP1/DAI) and cGAMP synthase (cGAS) signaling that ultimately activates IRF-3-dependent type I IFN response. KSHV encodes a viral interferon regulatory factor called vIRF1, targets STING by preventing it from interacting with TANK binding kinase 1 (TBK1), thereby inhibiting STING's phosphorylation and concomitantly activation, resulting in an inhibition of the DNA sensing pathway (Ma et al., 2015). By directly binding to cGAS, LANA encoded by KSHV inhibits the cGAS-STING–dependent phosphorylation of TBK1 and IRF-3, and thereby antagonizes the cGAS-mediated restriction of KSHV lytic replication (Zhang et al., 2016). On the other hand, production of KSHV *ORF52*, an abundant gammaherpesvirus-specific tegument protein, also subverts cytosolic DNA sensing by directly inhibiting cGAS enzymatic activity and reducing the dimerization and phosphorylation of IRF-3 (Wu et al., 2015). More interestingly, in addition to viral DNA, the DNA virus encoded RNAs can also be recognized by RNA sensors such as RIG-I, Toll-like receptor 3 (TLR-3), which mediates the type I IFN pathway against viral infection. The gammaherpesviruses, including EBV, KSHV and MHV68, each encode at least 12 microRNAs (miRNAs)

Figure 2. A schematic illustrating γ-herpesvirus mediated regulation of cellular protein phosphorylation to escape host innate immune surveillance.

(Feldman et al., 2014). EBV encoded small RNAs (EBERs) can be recognized by RIG-I and TLR-3 and induce downstream signaling pathways including phosphorylation of NF-κB and IRF-3, and release of interleukins 6 and 10 (IL-6/10) which act as cellular growth factors (Samanta et al., 2006; Munz, 2015; Iwakiri, 2016).

## Phosphorylation-mediated viral regulation of Interferon Regulator Factors

Upon PRR signaling by the IκB kinase (IKK)-related kinase IKKε and TBK-1, the phosphorylation of IRF-3 and IRF-7 are critical for IRF-3 homodimerization and translocation into the nucleus, where its interacts with the histone acetyl transferases CBP and p300, and associates with the IFN-β promoter. During the KSHV latent infection, to down-regulate expression of IKKε, it has been found that KSHV inhibits IKKε signaling by encoding viral miR-K12-11, and inhibiting IRF-3 phosphorylation in responsible for IRF-3 activation (Liang et al., 2011). Distinct from both KSHV and MHV-68 encoded ORF36 can only bind to phosphorylated IRF3, and inhibits the production of IFN-β (Hwang et al., 2009), EBV encoded ORF36 (namely BGLF4 kinase) could phosphorylates IRF-3 and inhibits the active IRF-3 recruitment to ISREs and thus suppresses the type I IFN response (Wang et al., 2009). Similar to IRF-3, IRF-7 is also phosphorylated by TBK1 and IKKε, which leads to heterodimerize with IRF-3 and fully stimulate type I IFN expression (Ning et al., 2011). It has been shown that although ORF45 of KSHV could inhibit IRF-7 phosphorylation (Zhu et al., 2002; Liang et al., 2012), the latent membrane protein LMP1 induces the phosphorylation and K63-linked ubiquitination of IRF7, resulting in its nuclear translocation and increased transcriptional activity (Ning et al., 2008; Bentz et al., 2012). Interestingly, LMP1 is also demonstrated to promote IRF-4 phosphorylation and markedly stimulate IRF-4 transcriptional activity (Wang et al., 2016).

## Phosphorylation-mediated viral regulation of ISG secretion and activation

It is well known that interferon binding to its receptor IFNAR could activate the Janus family protein kinases (JAKs) Tyk2 and Jak1, inducing site specific phosphorylation of tyrosine residues in the signal transducers and activator of transcription STAT1 and STAT2, leading to their activation and formation of a heterotrimeric complex containing IRF-9 (known as IFN- stimulated gene factor-3, ISGF3) (Taylor and Mossman, 2013). It has been found that each step of this interferon-mediated JAK/STAT signalling pathway is disrupted by herpes viral proteins (Figure 3). For examples, EBV-encoded the two latent membrane proteins LMP2A and LMP2B attenuate interferon responses by targeting the IFNARs and reducing JAK/STAT1 phosphorylation (Shah et al., 2009). The EBV immediate-early protein, BZLF1, can also decrease expression of the IFN-γ receptor and inhibit IFN-γ-induced STAT1 tyrosine phosphorylation and nuclear translocation, suggesting a mechanism by

Figure 3. Schematic representation of cytokine-mediated phosphorylation of JAK-STAT signaling pathways manipulated by γ-herpesvirus.

which EBV may escape antiviral immune responses during primary infection (Morrison et al., 2001). In contrast, KSHV K3 and K5 target the proximal IFN signal transduction by downregulating IFN-γR1 surface expression which led to effective suppression of IFN-γ-mediated STAT1 phosphorylation and transcriptional activation (Li et al., 2007). ORF54, a functional dUTPase from MHV-68, causes the degradation of the IFNAR1 protein independently of its dUTPase enzyme activity, and that this degradation results in a reduction of the type I IFN response, including the phosphorylation of STAT1 (Leang et al., 2011).

In addition, EBV LMP-1 could prevent Tyk2 phosphorylation and inhibits IFN-α-stimulated STAT2 nuclear translocation and its downstream genes transcription (Geiger and Martin, 2006). LMP1-induced tyrosine phosphorylation of STAT1 is almost exclusively due to the NF-κB-dependent secretion of IFNs. However, it remains to be further elucidated whether this response (which is usually considered to be antiviral) is in fact required for the EBV viral persistence (Najjar et al., 2005). Different from EBV, KSHV *ORF10* encodes a protein called RIF and forms a complex with Jak1, Tyk2, STAT2, and IFNAR subunits. Such complex appears to block activation of both Tyk2 and Jak1, as well as subsequent phosphorylation and activation of STAT1 and STAT2, with the consequence of failure of ISGF3 accumulation in the nucleus (Bisson et al., 2009). Moreover, KSHV vIRF-2 attenuates the accumulation of two components of ISGF-3: IRF-9 and phosphorylated STAT1, to inhibit type I IFN response (Mutocheluh et al., 2011).

Once activated, ISGF3 binds to the promoters and activates the expression of many IFN- stimulated downstream genes (ISGs) including genes expressing antiviral proteins. To date, three antiviral pathways have been well documented: the protein kinase R (PKR), the 2-5 OAS/RNaseL system and the Mx proteins (Haller et al., 2006). Additional ISG key proteins with potentially antiviral activities are ISG20, promyelocytic leukemia protein (PML), guanylate-binding protein 1 (GBP-1), p56 and RNA-specific adenosine deaminase 1 (ADAR1) (Haller et al., 2006). Among these, PKR as a serine/threonine kinase is also activated by dsRNA binding, and in turn exerts the effect on subsequent autophosphorylation and induction of apoptosis. Both KSHV and EBV individually encode EBERs and vIRF2 to bind PKR and inhibit itself phosphorylation, and thereby prevent IFN-α-mediated apoptosis (Burysek and Pitha, 2001; Samanta and Takada, 2010). Similarly, accumulating evidence indicates that PML-nuclear bodies (PML-NBs), also known as nuclear domain 10 (ND10) or PML oncogenic domains (PODs), is targeted by herpesviruses for viral transcription and replication (Chang et al., 2016). In NPC cells, it has been shown that EBNA1 disrupts PML-NB by inducing the degradation of PML proteins, leading to impaired DNA repair and increased cell survival. Further studies reveal that the interaction between EBNA1 and the cellular CK2 kinase is the key, and EBNA1 increases the association of CK2 with PML proteins, and thereby induces the phosphorylation and ubiquitylation of PML proteins for degradation (Sivachandran et al., 2010).

## Phosphorylation-mediated viral inhibition of NF-κB and inflammation

In resting cells, the inflammation responder NF-κB is held as an inactive complex in the cytoplasm by its inhibitor, IκBα. PRR activation stimulates IκBα phosphorylation and degradation, releasing NF-κB to translocate to the nucleus and induce target genes (Taylor and Mossman, 2013). Inhibition of NF-κB is employed by viruses as an immune evasion strategy which is also closely linked to oncogenesis during gammaherpesvirus persistent infection. For human KSHV and murine γHV68, NF-κB activation is sufficient to inhibit RTA-dependent transcriptional activation. Conversely, RTAs of KSHV and γHV68 were also shown to induce RelA (p65, the major component of NF-κB) degradation for blocking NF-κB activation. This likely contributes to the efficient lytic replication of γHV68 via evasion of antiviral cytokine production (He et al., 2014). For KSHV, it not only encodes vIRF3 to regulate the host immune system and apoptosis via inhibition of NF-κB activity by reducing IκB phosphorylation (Seo et al., 2004) during latent phase, but also encodes vGPCR (viral G protein-coupled receptor) during lytic replication to interact with and activate IKKε to promote NF-κB subunit RelA (p65) phosphorylation, which correlated with NF-κB activation and inflammatory cytokine expression (Wang et al., 2013). In addition, KSHV encoded K15 directly recruits NF-κB-inducing kinase NIK and induces NIK-mediated NF-κB p65 phosphorylation on Ser536 (Havemeier et al., 2014), while KSHV encoded vFLIP (a viral FLICE inhibitory Protein, encoded by the open reading frame K13) activates the NF-κB pathway by binding to NEMO, which results in the recruitment of IKK1/IKKα and IKK2/IKKβ and their subsequent activation by phosphorylation (Matta et al., 2012).

To block the activation of NF-κB-mediated inflammation pathway, EBV encoded LMP1 not only induces IκBα phosphorylation and degradation (Gewurz et al., 2011; Ersing et al., 2013), but also promotes RelA's (p65) phosphorylation and nuclear translocation (Zheng et al., 2007). Moreover, the key latent antigen called EBNA1 is also shown to inhibit the canonical NF-κB pathway in carcinoma lines by inhibiting the phosphorylation of IKKα/β. The reduction of both IκBα and p65 phosphorylation lead to decreased amount of p65 within the nuclear NF-κB complexes (Valentine et al., 2010). In the EBV-infected lymphoma cells, EBNA2 is specifically associated with upregulation of the two chemokines CCL3 and CCL4, which enhance Btk and NF-κB phosphorylation, and contribute to doxorubicin resistance of B lymphoma cells (Kim et al., 2016). Given the role of the EBV encoded BGLF4 as a potent viral kinase, it has been revealed to phosphorylate UXT (an NF-κB coactivator) at the Thr3 residue for blocking the interaction between UXT and NF-κB, and in turn reducing activity of NF-κB-mediated enhanceosome (Chang et al., 2012).

## Viral regulation of ATF2/c-Jun by phosphorylation

The AP-1 (activator protein 1) transcription factor is a dimeric complex that comprises several members including JUN (c-Jun, JunB, and JunD), FOS (c-Fos, Fos B, Fra1, and Fra2), ATF (activating transcription factor) and MAF (musculo-aponeurotic fibrosarcoma) protein families. AP-1 proteins are primarily considered to be oncogenic, and take part in a wide range of cellular events including cell transformation, proliferation, differentiation and apoptosis. Although much less is understood about ATF2/c-Jun, it has been documented that the c-Jun N-terminal kinases (JNKs) is activated, and then translocates into the nucleus for phosphorylating Jun proteins in a similar way to the activation process of NF-κB (Zheng et al., 2007). In NPC cells, EBV LMP1 could promote the formation of c-Jun/JunB heterodimers through inducing phosphorylation of c-Jun at ser63 and ser73. This heterodimeric form can bind to the AP-1 consensus sequence. The interaction between c-Jun and Jun B increases the repertoire of LMP1 regulatory complexes that could play an important role in the transcriptional regulation of specific cellular genes in the development of nasopharyngeal carcinoma (Song et al., 2004). In addition, given the role of EBV immediate-early protein BZLF1 (Zta) and BRLF1 (Rta) (two important regulatory factors for reactivation of lytic replication), both Zta and Rta activate the cellular stress mitogen-activated protein (MAP) kinases, p38 and JNK, resulting in phosphorylation (and activation) of the cellular transcription factor ATF2 (Adamson et al., 2000). In contrast, KHSV encoded lytic protein Orf49 could induce phosphorylation and activation of the transcription factor c-Jun, JNK and p38, suggesting that Orf49 activates the JNK and p38 pathways during the KSHV lytic cycle, and at least associates with cell proliferation, differentiation, or apoptosis (Gonzalez et al., 2006).

## Virus induces cytokines via phosphorylation-mediated activation of JAK-STAT

Cytokines play a critical role in many viral infections. Cytokine-mediated JAK/STAT signaling controls numerous important cellular processes, such as immune response, cellular growth, and differentiation. Viruses not only manipulate host cytokine production to favor virus survival, replication, and infection but also help virus-infected cells to escape the host immune response, which potentially results in the development of cancer (Wang et al., 2015). In the recent review (Wei et al.,

2016), we have summarized to address how EBV and KSHV encode their own cytokines and chemokines to escape hose immune surveillance. To avoid the redundancy, we will focus and highlight how viral phosphorylation manipulates production of cytokine through targeting JAK-STAT pathway (Figure 3). In addition to the IFN-associated JAK-STAT pathway, KSHV and EBV have been found to evade the immune response through expressing their own cytokines like vIL-6 and vIL10, respectively (Cai et al., 2010b; Cousins and Nicholas, 2013).

STAT3 as a major downstream target of the interleukin-6 (IL-6) and IL-10 families of cytokines, has been targeted by KSHV and EBV to induce many gene products, such as KSHV vIL-6 (Giffin et al., 2014), kaposin B (King, 2013), and viral-G-protein-coupled receptor (vGPCR) (Burger et al., 2005), miR-K12-1 (Chen et al., 2016) and EBV LMP-1 (Chen et al., 2003). By using the different binding receptor from the cellular homolog, vIL-6 could activate tyrosine phosphorylation of STAT3 via gp130-associated JAK pathways and MAPK serine/threonine kinase pathways (Cai et al., 2010b; Cousins and Nicholas, 2013). Meanwhile, the phosphorylation levels of STAT3 influence viral lytic reactivation in cell culture (Reddy et al., 2016). STAT3 contributes to maintenance of latency by curbing lytic activation of EBV and KSHV in latent cells that express high levels of STAT3. While activated STAT3 plays a key role in suppressing the DNA damage response, which facilitates cell proliferation as well as development of cancer (Li and Bhaduri-McIntosh, 2016). In the MHV68 animal model, it has been shown that STAT3 expression is also required for virus to establish latency in primary B cells under the circumstance of active immune response to infection (Reddy et al., 2016).

STAT6 is another major downstream target of transcriptional factor activated by cytokine IL-4 or IL-13, and has been demonstrated to target by EBV and KSHV. Selectively activation of IL-4/STAT6 and IL-13/STAT6 signaling is utilized by KSHV to promote pathogenesis and tumorigenesis during latency infection (Cai et al., 2010a; Wang et al., 2017). Among these, LANA encoded by KSHV is essential for viral blocking of IL-4-induced signaling. LANA reduces IL-4-mediated phosphorylation of STAT6 on Y-641 and concomitantly its DNA binding ability (Cai et al., 2010a). However, STAT6 is constitutively activated in the PEL cells due to the secretion of IL-13 and downregulation of SHP1 by KSHV (Wang et al., 2015; Wang et al., 2017). Moreover, IL-13-stimulated constitutively phosphorylation of STAT6 is tightly associated with activation of JAK1 instead of PI3K and Akt (Wang et al., 2015). However, both IL-4 and IL-13 also activate STAT6 and induced by LMP-1 in EBV-infected B cells (Kis et al., 2011).

## FUTURE PERSPECTIVES

In the view of the fact that phosphorylation events involve in viral replication, immune evasion and oncogenesis, the cellular and viral kinases utilized by EBV and KSHV may serve as targets for prophylactic and therapeutic treatments of viral infections. Some kinase-inhibitory compounds, including nucleoside analogues, tyrosine kinase inhibitor, Serine Threonine kinase inhibitor, have previously been successful in treating various cancers, and researches are ongoing to determine their efficacies against viral infections (Keating and Striker, 2012).

To date, a number of nucleoside analogues, such as acyclovir and ganciclovir, have been developed in order to exploit these viral kinases for therapeutic purposes. These nucleoside analogues are first phosphorylated by viral kinases and subsequently phosphorylated by cellular kinases to form nucleoside triphosphates. In general, the nucleoside analogues will inhibit viral DNA replication and thus decrease herpes viral replication (Keating and Striker, 2012). Among these, the potential development of tyrosine kinase inhibitors as a safe and effective prophylactic and therapeutic treatment against poxviruses is a point of interest in current research (Schang, 2006), including the Abl- and Kit-specific imatinib which selectively inhibits the tyrosine kinases Abl and c-kit (Koon et al., 2005). Taking these concepts even further, Koon and colleagues demonstrated that imatinib is also active against the pathogenesis of KSHV-induced Kaposi's sarcoma (Schang, 2006). The two serine/threonine kinase inhibitors of the cellular mTOR kinase, Sirolimus and everolimus (rapamycin), have been shown to successfully treat some of Kaposi's Sarcoma (KS) but not all in the transplant patients (Campistol et al., 2004; Stallone et al., 2005; Stallone et al., 2008). In addition, some NF-κB inhibitors have also presented antiviral and antitumor function. For instances, the NF-κB inhibitor BAY11-7082 could induce PEL cells apoptosis, and Diallyl trisulfide (DAT) could suppress the production of viral progeny from PEL cells (Shigemi et al., 2016). Celastrol, a TAK1/NF-κB inhibitor, has also presented as a potential therapeutic molecule to ameliorate vGPCR/KSHV-induced tumors (Bottero et al., 2011). As an inhibitor of Pim kinases, the chemical compound tricyclic benzo and its derivative were shown to dramatically reduce the motility and proliferation of EBV transformed lymphoblastoid cells (Kiriazis et al., 2013). Similar effect was also observed on KSHV-induced lymphoma cells (Sarek et al., 2013). In summary, based on the recent understanding of molecular regulatory modes of phosphorylation events targeted by EBV and KSHV, and their potential chemical compounds discovered, we believe that safe and effective prophylactic and therapeutic strategies against their related cancers will be achieved in near future.

## ACKNOWLEDGMENT

The authors would like to apologize to the many researchers who have contributed to this area of research but have not been cited in this review due to space limitations. This work is supported by the National Natural Science Foundation of China (NO. 81471930, 81402542, 81672015, 81772166), and National Key Research and Development Program of China (2016YFC1200400). FW is a scholar of Pujiang Talents in Shanghai. QC is a scholar of New Century Excellent Talents in University of China.

## COMPLIANCE WITH ETHICS GUIDELINES

The authors declare that they have no conflict of interest. This article does not contain any studies with human or animal subjects performed by any of the authors.

## OPEN ACCESS

This article is distributed under the terms of the Creative Commons Attribution 4.0 International License (https://creativecommons.org/licenses/by/4.0/), which permits unrestricted use, distribution, and reproduction in any medium, provided you give appropriate credit to the original author(s) and the source, provide a link to the Creative Commons license, and indicate if changes were made.
